# Auxin-Abscisic Acid Interactions in Plant Growth and Development

**DOI:** 10.3390/biom10020281

**Published:** 2020-02-12

**Authors:** Ryan J. Emenecker, Lucia C. Strader

**Affiliations:** 1 Department of Biology, Washington University, St. Louis, MO 63130, USA; remenecker@wustl.edu; 2 Center for Science and Engineering Living Systems (CSELS), Washington University, St. Louis, MO 63130, USA; 3 Center for Engineering Mechanobiology, Washington University, St. Louis, MO 63130, USA

**Keywords:** auxin, abscisic acid, phytohormone, plant development, phytohormone interactions

## Abstract

Plant hormones regulate many aspects of plant growth, development, and response to biotic and abiotic stress. Much research has gone into our understanding of individual plant hormones, focusing primarily on their mechanisms of action and the processes that they regulate. However, recent research has begun to focus on a more complex problem; how various plant hormones work together to regulate growth and developmental processes. In this review, we focus on two phytohormones, abscisic acid (ABA) and auxin. We begin with brief overviews of the hormones individually, followed by in depth analyses of interactions between auxin and ABA, focusing on interactions in individual tissues and how these interactions are occurring where possible. Finally, we end with a brief discussion and future prospects for the field.

## 1. Introduction

Phytohormones profoundly affect plant growth and development. Decades of research have elucidated the molecular underpinnings of many phytohormones, including their biosynthetic pathways, primary signaling components, and their transcriptional outputs. Whereas this work has revealed remarkable complexities within individual phytohormone pathways, connections among the pathways are less well defined. In contrast to the classical view in which each hormone regulates a set of discrete processes or facilitates responses to specific stimuli, evidence suggests the various phytohormones function as an interconnected network working together to regulate plant growth and development. At its core, these interactions describe the phenomenon whereby one hormone directly affects an aspect of a different hormone to coordinately regulate a specific process. This can be through directly altering the abundance of another hormone or by changing the abundance or activity of components involved in the signaling of another hormone. 

Whereas our understanding of the activity of individual phytohormones is comparatively advanced, our molecular understanding of how phytohormones influence each other and how this ultimately results in the regulation of various processes is less complete. Unraveling the complexities of phytohormone interactions is by no means a trivial matter. Multiple plant hormones are typically involved in regulating each developmental event and response to stimuli. The various phytohormones clearly work together in an interconnected network to respond to stimuli and control growth and developmental processes. This interconnectedness has important implications for our interpretation of data using hormone mutants, and for our understanding of regulation of growth responses. In this review, we focus on interactions between two of the classical plant hormones, auxin and abscisic acid (ABA).

Auxin is remarkable in that it is seemingly involved in the regulation of most plant growth and developmental responses [1]. Furthermore, auxin transport and response are frequently implicated in interactions with other plant hormones [2,3,4,5,6]. At its most fundamental level, auxin affects plant growth and development through regulation of cell division and expansion [7]. Whereas auxin has classically been considered a “growth hormone”, ABA is frequently defined as a “stress hormone” with roles in the regulation of biotic and abiotic stress responses [8]. Despite these classic definitions, ABA plays roles in plant growth and development under non-stress conditions [9] and auxin plays roles in response to various stress stimuli [10]. In addition, ABA and auxin pathways interact in regulation of various growth and stress responses [11]. Before diving into interactions between auxin and ABA, we will briefly review first auxin and then ABA homeostasis and response followed by in-depth analysis of how interactions between these two hormones govern various distinct growth processes. Finally, we will end this review with a brief discussion and future directions for understanding auxin and ABA interactions.

## 2. Auxin Homeostasis and Response

### 2.1. Auxin Biosynthesis

**Figure 1:**
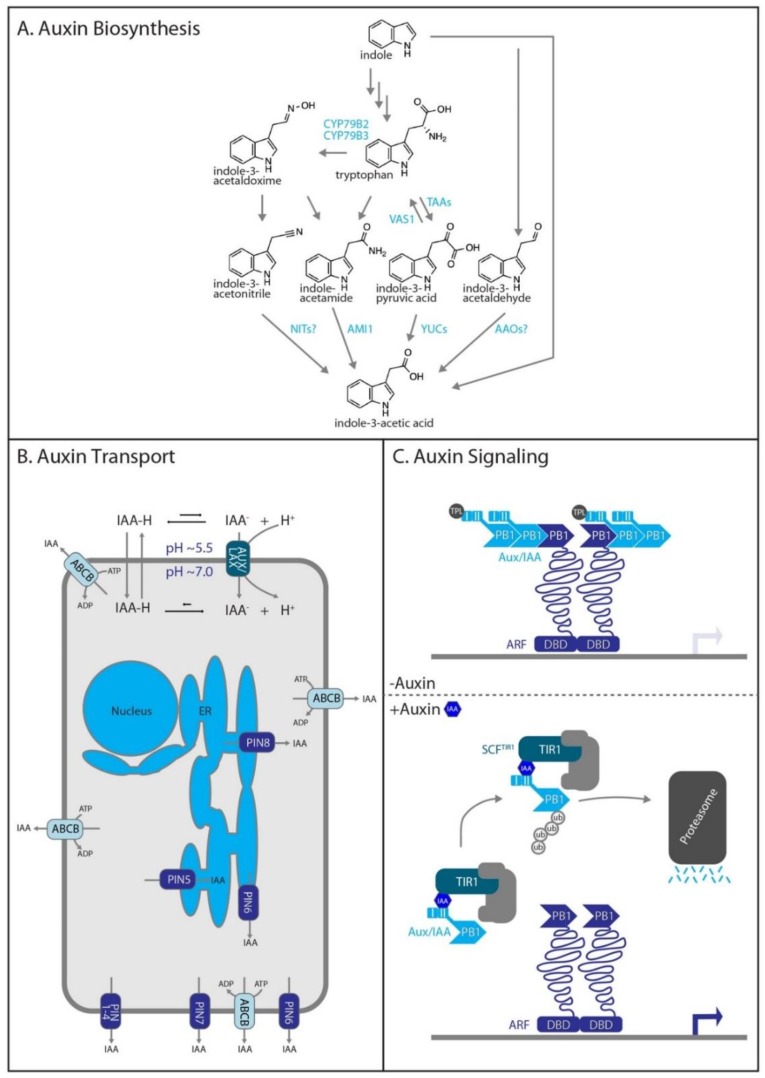
Auxin biosynthesis, transport, and signaling. (**A**) Auxin biosynthesis. Indole contributes to tryptophan-dependent and tryptophan-independent auxin biosynthetic pathways. (**B**) Auxin Transport. Three major transporters are included in this figure, the PIN family, the ABCB family, and the AUX1/LAX family. Direction of auxin transport for each of the transporters is indicated by the arrow through the transporter. PIN6 is being shown as both in the ER and the plasma membrane as dual localization of PIN6 has been demonstrated [25]. (**C**) Auxin signaling. In the absence of auxin (top), the Aux/IAA family of repressors interacts with and represses the ARF family of transcription factors. However, in the presence of auxin (bottom), the Aux/IAA repressors are ubiquitinated by an SCF complex and targeted for degradation by the 26S proteasome. This relieves the ARF transcription factors from repression, allowing them to carry out a transcriptional response.

The predominantly studied bioactive form of auxin is indole-3-acetic acid (IAA). Auxin homeostasis is regulated by multiple inputs into the pool of free IAA (reviewed in [12]). The indole-3-pyruvic acid (IPyA) pathway appears to be the main contributor of free IAA [13]. The first step of the pathway consists of the conversion of tryptophan to IPyA by the Tryptophan Aminotransferase of Arabidopsis (TAA) family of tryptophan aminotransferases. This is followed by conversion of IPyA to IAA via the Yucca (YUC) family of flavin monooxygenases (Figure 1a). In addition to the major route to auxin through IPyA, IAA can be synthesized from indole-3-acetonitrile, indoleacetamide, indole-3-acetylaldehyde, as well as from the chain-lengthened precursor indole-3-butyric acid (IBA), sugar conjugates, and amino acid conjugates [12]. Distinct developmental events and stimuli may differentially affect IAA levels through modulation of one or more of these pathways [14]. For example, high temperatures have been shown to result in increased levels of free IAA, resulting in hypocotyl elongation [10]. Similarly, the shade avoidance response has been shown to specifically require an intact IPyA biosynthesis pathway [15]. For in-depth reviews on auxin biosynthesis, please see [12,16].

### 2.2. Auxin Transport

Proper auxin transport is required for organ development, root branching, shoot branching, phototropism, and gravitropism [17]. Cellular auxin uptake is in part possible through the differences in pH between the cytosol and the apoplast of plant cells. Under normal growth conditions, the apoplast is mildly acidic (pH ~ 5) [18]. Under these conditions in the apoplast, a small percentage of auxin exists in its protonated form, which is able to diffuse across the plasma membrane and into the cell. In addition, auxin is moved from the apoplast to the cytoplasm by the Auxin Resistant 1/ Like Aux1 (AUX1/LAX) family of transporters [17]. The cytoplasmic pH is neutral (pH ~ 7). Under these less acidic conditions, cytoplasmic auxin exists almost entirely in its deprotonated form, which is unable to passively diffuse across the plasma membrane. Thus, while some amount of auxin uptake can occur passively, auxin efflux depends almost entirely on specific transporters. Two main families of transporters export auxin from the cell, the Pin-formed (PIN) family and the type B ATP-Binding Cassette (ABCB) transporters. The PINs, specifically PIN1-4 and PIN7 display polar plasma membrane localization, largely underly polar auxin transport [19] and are required for directional movement of auxin. In addition to these plasma membrane-localized transporters, auxin transporters on the ER, such as the majority of the PIN-Likes (PILs) family, and the vacuole play roles in auxin homeostasis [20] (Figure 1b). For in-depth reviews on auxin transport, please see [17,20].

### 2.3. Auxin Signaling

Auxin-regulated transcriptional responses are controlled by the nuclear auxin signaling pathway. The pathway consists of the E3 ubiquitin ligase complex SCF^TIR1/AFB^ (TRANSPORT INHIBITOR RESPONSE 1/AUXIN-RELATED F-BOX PROTEINS), the Auxin/Indole-3-Acetic Acid (Aux/IAA) repressor proteins, and the Auxin Response Factor (ARF) transcription factors. Briefly, activity of the ARF transcription factors is repressed by interaction with an Aux/IAA protein. However, auxin promotes interaction of Aux/IAA repressor proteins with the TIR1/AFB F-box component of the SCF^TIR1/AFB^ ubiquitin E3-ligase complex, leading to Aux/IAA polyubiquitylation and degradation by the 26S proteasome. This removal of Aux/IAA repressor proteins from the system frees members of the ARF family to mediate auxin-responsive transcriptional responses (Figure 1c). Please see [21,22,23,24] for detailed reviews of this pathway.

## 3. ABA Homeostasis and Response

### 3.1. ABA Biosynthesis

**Figure 2:**
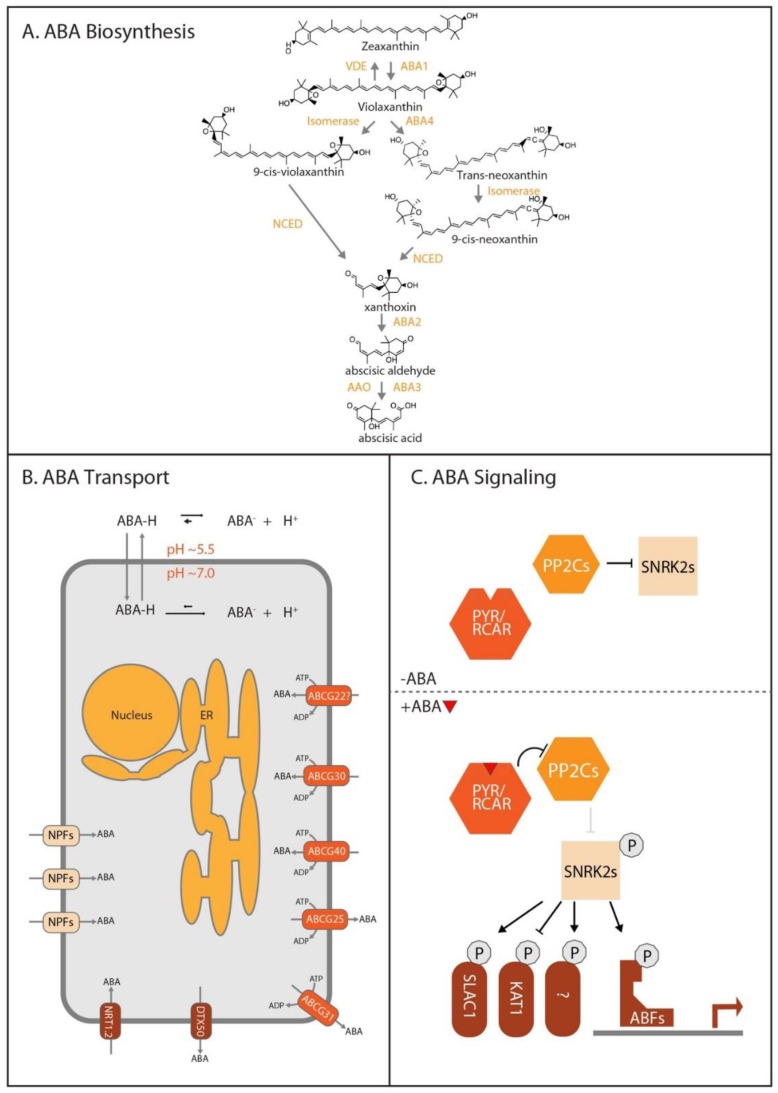
ABA biosynthesis, transport, and signaling. (**A**) ABA biosynthesis. ABA biosynthesis begins with isoprenoids derived from the plastidic methyl-D-erythritol-4-phosphate (MEP) pathway, ultimately leading to the carotenoid zeaxanthin. Zeaxanthin then feeds into the ABA biosynthetic pathway. (**B**) ABA transport. Direction of ABA transport for each of the transporters is indicated by the arrow through the transporter. ABCG22 is listed with a question mark due to it currently not being directly shown to transport ABA but was included due to evidence suggesting that it is involved in ABA transport. Similarly, multiple NRT1/PTR (NPF) family transporters are simply designated as “NPFs?” due to their current standing as possibly involved, but no direct evidence with the exception of NRT1.2 (also known as NPF4.6) has been provided for their involvement in ABA transport. (**C**) ABA signaling. In the absence of ABA (top), the PP2C protein phosphatases interact with and represses the SnRK2 kinases through dephosphorylation. However, in the presence of ABA (bottom), PYR/PYL/RCAR ABA receptors inhibit the PP2Cs, relieving SnRK2 kinases from repression. This leads to SnRK2 phosphorylation and activation, allowing SnRK2s to phosphorylate downstream targets such as the ABRE-binding factors (ABFs) transcription factors.

ABA is a small sequisterpene derived from carotenoids, and thus its biosynthesis begins with isoprenoids derived from the plastidic methyl-D-erythritol-4-phosphate (MEP) pathway [26]. The specific carotenoid that leads to ABA biosynthesis is zeaxanthin. The zeaxanthin epoxidase ABA Deficient 1 (ABA1) converts zeaxanthin into violaxanthin; this step is reversible and thus not a committed step in the ABA biosynthetic pathway. Zeaxanthin and violaxanthin can be converted into 9′-cis-neoxanthin and 9′-cis-violaxanthin, respectively. Conversion of 9′-*cis*-neoxanthin and 9′-*cis*-violaxanthin into xanthoxin through the 9-cis-epoxycarotenoid dioxygenase (NCED) family of enzymes represents the first committed step to ABA biosynthesis and is thought to be rate limiting [27]. Xanthoxin is converted to abscisic aldehyde through the enzymatic action of ABA Deficient 2 (ABA2) [28]. Abscisic aldehyde is used by enzymes in the Abscisic Aldehyde Oxidase (AAO) family, primarily AAO3 [29], to produce abscisic acid (Figure 2a). *NCED* gene transcription is differentially regulated by various stimuli, underscoring NCED roles in context-specific ABA biosynthesis. For in-depth reviews of ABA biosynthesis, please see [26,30].

### 3.2. ABA Transport

Plants primarily synthesize ABA in the vasculature and guard cells of vegetative tissue [31]. Further, experimental data suggests that in response to drought, most ABA found in the roots is synthesized in the shoots [32]. These data suggest that ABA transport from the shoots to the roots may be critical to proper ABA response. However, few ABA transporters have been identified, possibly due to genetic redundancy. Genetic evidence and heterologous expression studies have resulted in the identification of three ABA exporters: Arabidopsis ATB-Binding Cassette G25 (AtABCG25) [33], AtABCG31 [14], and Detoxification Efflux Carrier 50 (AtDTX50) [34] and three ABA importers: AtABCG30 [14], AtABCG40 [35], and AtNRT1.2 [14,31,34] (Figure 2b). Further, AtABCG22 may have a role ABA transport, but further studies are needed to confirm this [36]. For in-depth reviews of ABA transport, please see [31,37,38].

### 3.3. ABA Signaling

ABA is perceived by the 14-member Pyrobactin Resistance/Pyrobactin 1-Like/Regulatory Components of ABA Receptor (PYR/PYL/RCAR) family of proteins [39]. Binding of ABA by PYR/PYL/RCAR family members results in inhibition of the 9-member clade A Protein Phosphatase 2Cs (PP2Cs) [40], which act as negative regulators of ABA response. These PP2Cs repress the Slow Anion Channel Associated 1 (SLAC1) ion channel [41] and Sucrose Nonfermenting1-Related Protein KinasE2 (SnRK2) kinases [42].

Thus, PP2C inhibition by ABA-mediated interaction with PYR/PYL/RCAR members allows for SLAC1 and SnRK2 family activity. Active SnRK2 kinases phosphorylate and regulate various targets involved in ABA response including transcription factors [43] and ion channels such as the Potassium Channel in *Arabidopsis thaliana* 1 (KAT1) [44] (Figure 2c). Thus, major outputs of ABA signal transduction are altered gene expression and altered ion channel activity. For an in-depth review of the ABA signaling pathway, please see [45].

## 4. Auxin-ABA Interactions

The effects of auxin and ABA on various growth processes has been well-documented (Table 1). Further, through analysis of the responses of various ABA and auxin mutants, how these interactions work on a molecular basis is beginning to be better understood (Table 1). Notably, auxin tends to act downstream of ABA in regulation of many examined processes. Because auxin regulates growth through cell elongation and division, auxin action downstream of ABA to regulate growth processes is logical (reviewed in [7]). In the future, a comprehensive analysis of the auxin responsiveness of ABA biosynthesis, transport, and signaling mutants will be required to determine whether ABA acts downstream in any auxin-regulated process.

ABA-auxin interactions may vary in a tissue-dependent fashion. By analyzing the effect of auxin or ABA on various auxin or ABA biosynthesis, transport, or signaling mutants, we can begin to determine which components are necessary for response to the other hormone. In this review, we focus on auxin-ABA interactions governing seed germination, hypocotyl elongation, root elongation, lateral root formation, and cotyledon expansion.

### 4.1. Auxin -ABA Interactions in Seed Germination

Seed germination begins with imbibition of dry seed followed by emergence of the embryonic root, which is called the radicle. The molecular mechanisms underlying seed germination in Arabidopsis are relatively well understood, and ABA is known to be integral in the regulation of seed dormancy and therefore timing of seed germination [62]. For a review on the molecular mechanisms underlying seed germination, please see [63]. Strong genetic evidence supports a model whereby ABA-mediated inhibition of seed germination requires intact auxin biosynthesis, transport and signaling. With respect to auxin biosynthesis, the *yuc1 yuc6* [48] mutant, defective in the IPyA pathway, displays premature germination and mild resistance to the inhibitory effects of ABA on seed germination [55]. Conversely, auxin overproduction results in hypersensitivity to ABA in germination inhibition assays [55,64]. Furthermore, auxin enhances the inhibitory effects of ABA in germination assays. These data suggest a model whereby auxin homeostasis is downstream of ABA in regulation of seed germination [55].

Auxin transport is also required for the inhibitory effects of ABA on seed germination. In a forward genetics screen looking to isolate mutants resistant to exogenous ABA in root elongation assays, Thole et al., 2014 [51], identified a new allele of the auxin influx transporter AUX1, which had previously been identified as resistant to ABA in root elongation assays [65], as resistant to ABA in seed germination inhibition assays [51]. Furthermore, the authors identified an allele of Ethylene Insensitive Root1/PIN-Formed2, a known auxin efflux carrier, as resistant to ABA in seed germination assays [51]. Similar resistance of PIN2 mutants to abscisic acid in seed germination had also been reported previously [66]. This suggests that both functional auxin influx as well as auxin efflux is necessary for ABA to exert its inhibitory effects on seed germination.

Auxin signaling acts downstream of ABA in the inhibition of seed germination. Mutants defective in genes encoding the auxin receptor *TIR1* [51], the dual-specificity protein phosphatase *Indole-3-Butyric Acid Response5* (*IBR5*) [67], or the RUB modifying enzyme *Auxin Resistant1* (*AXR1*) [51,67] display resistance to the inhibitory effects of ABA on seed germination. Further, stabilizing mutations in Aux/IAA repressors, including *axr3-1I* [56], *axr2-1* [56], *slr1-1* [56], and *iaa16-1* [56], result in ABA-resistant seed germination. Multiple Auxin Response Factors (ARFs) have roles in ABA-mediated repression of seed germination. Mutants defective in *ARF10* or *ARF16* display resistance to ABA in germination assays [55] whereas mutants defective in *ARF2* display hypersensitivity to ABA in seed germination assays [68]. Thus, defects in multiple aspects of auxin signaling result in altered sensitivity to the inhibitory effects of ABA on seed germination.

Analysis of various auxin mutants in ABA-mediated germination inhibition assays results in a model in which intact auxin transport, biosynthesis, and signaling are necessary for full responsiveness to ABA in seed germination. However, auxin treatment does not inhibit seed germination like ABA, suggesting that ABA effects on seed germination are more complex than simply activating auxin. Because auxin enhances the propensity of ABA to inhibit seed germination, examination of various ABA biosynthesis, transport, and signaling mutants in seed germination assays using both auxin and ABA may help further tease apart the underpinnings of auxin-ABA interactions with respect to seed germination.

Although many auxin pathway components have been found to be necessary for inhibition of germination and post-germinative growth by ABA, the molecular underpinnings of this interaction are not understood. For example, does ABA inhibit these processes at least partially by upregulating auxin levels? By modifying players in the pathway (for example PIN localization or Aux/IAA stability)? Understanding exactly how this ABA-auxin interaction occurs is critical.

### 4.2. Auxin–ABA Interactions in Cell Expansion and Hypocotyl Elongation

Hypocotyl elongation is driven solely by cell expansion [69]. For a thorough review on these mechanisms see [70]. Plant cell expansion is governed by the acid-growth theory [71]. Broadly, auxin induces cell elongation through acidification of the cell wall by activation of plasma membrane H+-ATPases, which excrete protons into the apoplast. The reduced pH in the apoplast leads to a series of events that result in “loosening” of the cell wall. This loosening allows turgor pressure-driven irreversible expansion of the cell wall, ultimately resulting in cell elongation. Although the acid-growth theory is decades old, molecular mechanisms underlying how auxin promotes apoplast acidification have only recently been uncovered.

The plasma membrane H+-ATPase (PM-H+ATPase) *Arabidopsis* H+ATPase 2 (AHA2), ultimately regulates apoplastic pH [72] by pumping protons into the extracellular space. AHA2 activity is regulated by phosphorylation via an unknown kinase and dephosphorylation by members of the PP2C.D family of protein phosphatases. Phosphoactivation of AHA2 is dynamic and regulated by the plant hormones auxin and ABA.

**Figure 3:**
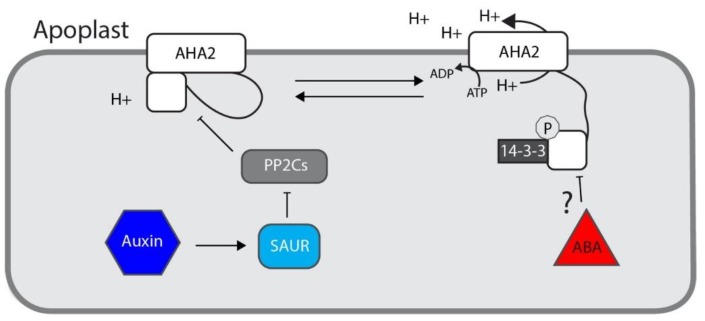
Auxin and ABA effects on AHA2 activity. Auxin promotes expression of *SAURs*. A subset of the SAURs inhibit the PP2C.Ds, ultimately relieving repression of PM-H+ATPases such as AHA2 and resulting in cell wall acidification. How ABA results in dephosphorylation of PM-H+ATPases is not known, but evidence suggests that ABA does directly result in dephosphorylation and inhibition of PM-H+ATPases [46] to affect cell wall acidification.

*Small Auxin-UP RNA* (*SAUR*) genes are direct primary targets of auxin signal transduction [73]. SAUR19 promotes cell expansion through derepression of AHA2. Specifically, SAUR19 binds to PP2C.D family members that target AHA2 [74], effectively repressing their activity and leading to elevated AHA2 phosphorylation activation (Figure 3). For further reading in this area please see references [75,76,77].

Treatment of either light-grown or dark-grown seedlings with ABA results in inhibition of hypocotyl elongation [48]. In contrast to ABA, treatment of light-grown seedlings with auxin results in increased hypocotyl lengths, whereas treatment of dark-grown seedlings with auxin results in decreased hypocotyl lengths. This difference is presumed to be due to different starting levels of auxin, although this has not been experimentally determined. In this model, light-grown seedlings likely accumulate low levels of hypocotyl auxin in the absence of environmental cues that promote hypocotyl elongation. Conversely, auxin and growth are maximized in dark-grown seedlings, which are driven to put their energy into elongating this organ until sunlight can be reached. Treatment of dark-grown seedlings with exogenous auxin therefore results in supra-optimal auxin levels, resulting in growth inhibition.

In dark-grown seedlings, both auxin and ABA inhibit hypocotyl growth. Exogenous ABA negatively impacts both the elongation rate and overall elongation of etiolated hypocotyls in a manner that requires an intact ABA signal transduction pathway, as a gain-of-function mutation in ABI1 results in ABA-resistant dark-grown hypocotyl elongation [46]. Furthermore, ABA inhibits the phosphorylation of plasma membrane H+ATPases [46]. Notably, specific ABI1 roles in this process and its relationship to the SAUR-PP2C.D regulation AHA2 are yet to be uncovered. Regulation of AHA2 activity provides an excellent potential point of auxin-ABA interaction in the regulation of dark-grown hypocotyl elongation; however, it is not yet known whether phosphoregulation of plasma membrane H+ATPases is the mechanism by which auxin inhibits dark-grown hypocotyl elongation.

Little is known about factors required for interaction between auxin- and ABA-responsive control of hypocotyl elongation. Exogenous ABA results in down-regulation of the auxin biosynthetic genes *YUC3*, *YUC5*, and *YUC6* in both dark-grown and red light-grown seedlings [48] suggesting that ABA may influence hypocotyl elongation by decreasing auxin levels; however, further analyses such as quantification of auxin levels in response to ABA will be necessary to determine if this is a mechanism of auxin-ABA interaction. Further, it should be noted that this expression analyses was performed with whole seedlings [48] and may not be an accurate representation of hypocotyl-specific differences in expression of auxin biosynthetic genes in response to ABA.

Our understanding of auxin-ABA interactions in the context of hypocotyl elongation is in its infancy. Elucidating how these two hormones interact to govern hypocotyl elongation will be important to understand how stress response and growth response are integrated for these two critical hormones during seedling establishment—we hypothesize that these pathways will converge upon regulation of AHA activity. Characterizing the auxin and ABA responsiveness of various auxin and ABA signaling, biosynthesis, and transport mutants will be important to lay the groundwork for our understanding of how these two hormones work together to regulate hypocotyl elongation.

### 4.3. Auxin–ABA Interactions in Root Elongation

Root elongation relies on a combination of cell division and cell elongation. Both auxin [78] and ABA [79] play integral roles in the regulation of root elongation. For a review on the molecular mechanisms underlying root elongation, please see [80]. High levels of exogenous auxin and abscisic acid inhibit root elongation [51]. Mutant screens for resistance to the inhibitory effects of auxin uncovered many auxin signaling mutants, but no mutants defective in the ABA pathway. Conversely, a screen for resistance to the inhibitory effects of ABA on root elongation uncovered mutants defective in both the ABA and the auxin pathways [51,65,67]. Specifically, disruption of auxin transport (*aux1* or *pin2;* [51]) or auxin signaling (*tir1*, *ibr5*, *axr1*, and gain-of function *Aux/IAA* mutants; [51,56]) result in resistance to ABA in primary root elongation assays. Further, defects in the Class-B “repressor” ARF2 lead to hypersensitivity to ABA in root elongation assays whereas ARF2 overexpression results in ABA resistant root elongation [68]. Thus, an intact auxin transport and signaling system is necessary for full responsiveness to ABA in root elongation assays.

A recent study investigating both low levels of exogenous ABA (0.1 µM), which can stimulate root growth, and the higher concentrations of exogenous ABA (10 µM), which inhibit root growth, demonstrates that auxin signaling and transport are necessary for both the stimulatory and the inhibitory effects of ABA on root growth [81]. Interestingly, auxin efflux is necessary for the stimulatory effects of low concentrations of ABA on root elongation whereas auxin influx is seemingly dispensable for these effects [81]. Conversely, both auxin efflux and auxin influx are necessary for the inhibitory effects of high levels of ABA on root elongation

ABA-mediated inhibition of primary root elongation clearly requires intact auxin transport and signaling. Conversely, auxin-mediated inhibition of root elongation does not require intact ABA signaling. ABA signaling mutants, including *abi1-1*, *abi2-1*, and *abi3-1*, display wild-type responsiveness to the synthetic auxin 2,4-D [51]. Thus, auxin acts downstream of ABA in regulation of root elongation. Although auxin response is required for the inhibitory effects of ABA on root elongation, how ABA affects auxin to elicit this response is unknown. Understanding the molecular underpinning of this interaction, including effects on auxin transport, biosynthesis, and signal transduction, will be critical for elucidating this process.

### 4.4. Auxin–ABA Interactions in Lateral Root Formation

Lateral root formation is an integral driver of overall root system architecture [82] and is critical for soil exploration and anchoring plants. Lateral roots initiate inside the primary root from xylem-pole-pericycle cells [82]. Lateral root formation is promoted by exogenous auxin and inhibited by exogenous ABA [58,59]. For a review on lateral root formation, please see [82].

ABA likely exerts its repressive effects on lateral root formation through auxin [83]. In particular, the ABA receptor PYL9 enhances transcriptional activity of MYB Domain Protein77 (MYB77) [84], an ARF7 interactor that regulates lateral root formation [85]. Thus, PYL9 through interaction with MYB77 may play a direct role in regulation of lateral root formation through auxin-ABA interactions.

Two additional components of the ABA signaling pathway have been implicated in regulation of lateral root formation through auxin-ABA interactions [61,86]. Specifically, *abi3* mutants are resistant to auxin in the promotion of lateral root formation [61]. Further, auxin inhibits *ABI3* expression [61]. In addition, ABI4 regulates lateral root formation [86]. Loss of ABI4 function leads to increased lateral root formation; conversely, *ABI4* overexpression results in fewer lateral roots [86]. ABI4 inhibits lateral root formation through reducing the level of the auxin efflux carrier PIN1, likely resulting in a reduction in polar auxin transport [86]. Because auxin and ABA are each critical to lateral root formation, particularly in response to soil water conditions, fully understanding this relationship is critical.

### 4.5. Auxin–ABA Interactions in Cotyledon Growth

Arabidopsis cotyledons appear to grow through a combination of both cell expansion and cell division [87], although cell expansion appears to be the strongest driver [88]. Endogenous auxin promotes cotyledon expansion [89]; however, treatment with either exogenous auxin or exogenous ABA results in decreased cotyledon area [48,57]. Gain-of-function mutations in *IAA7* (*axr2-1*), *IAA17* (*axr3-1*), or *IAA16* (*iaa16-1*) result in resistance to ABA in seed germination assays and to the inhibitory effects of ABA on post-germinative cotyledon expansion [56], suggesting that auxin acts downstream of ABA in this process. In depth analyses of auxin and ABA mutants in response to both auxin and ABA in regulating cotyledon expansion will be necessary to understand roles that auxin-ABA interactions play in governing cotyledon growth.

## 5. Discussion

ABA and auxin clearly interact extensively in the regulation of plant growth and development. Understanding these interactions will be fundamental to our ability to interpret experiments using mutants defective in either auxin or ABA homeostasis and response. Furthermore, by elucidating which aspects of auxin or ABA homeostasis are required for proper response to the other hormone, we may begin to uncover the molecular mechanisms by which these interactions occur. Whereas much has been done to elucidate auxin-ABA interactions in some developmental programs, such as in root elongation and seed germination, we do not yet understand this interaction in many other important growth processes. Although there are tantalizing hints that auxin-ABA interactions are important in hypocotyl elongation, lateral root formation, and cotyledon growth, a full understanding of these interactions is lacking. Simple growth assays, for which the necessary mutants are already available, to examine the nature of auxin-ABA interactions in these processes may help us elucidate the fundamental nature of auxin-ABA interactions in these contexts. For example, we may find that root elongation, which is a process driven by a combination of cell division and cell elongation, has a distinct mechanism underlying auxin-ABA interactions in comparison to something such as hypocotyl elongation, which is driven solely by cell expansion. Alternatively, we may find that the interactions between auxin and ABA are identical across growth processes.

Critically, a molecular understanding of the interactions between these pathways is lacking. For example, do ABA and auxin affect the other hormone’s homeostasis in any of the developmental contexts described above? Or are the interactions between these pathways enacted through modulation of sensitivity to the other hormone by affecting the activity or stability of signaling components? Alternatively, these pathways could converge upon shared sets of gene targets. Ultimately, teasing apart these molecular possibilities will be critical for understanding and manipulating auxin-ABA interactions.

## 6. Conclusions and Future Directions

Here, we synthesized available information on auxin-ABA interactions in regulation of specific growth processes. In addition to the aspects of ABA and auxin covered (biosynthesis, transport, signaling), there may be other possible points of auxin-ABA interaction, including regulation of hormone conjugation, degradation, and regulation of overlapping sets of genes. Future studies will be required to examine the importance of these various processes in auxin-ABA interactions.

Establishing which tissues and growth processes require intact auxin or ABA homeostasis for response to the other hormone in the future will be important for understanding the nature of interactions between these hormones. Reports to date suggest that auxin acts downstream of ABA to regulate the same process, no matter whether both hormones have the same effect or the opposite effect on a process. This raises the question of whether there are any contexts in which ABA acts downstream of auxin. Further, this trend also suggests that ABA may promote auxin activity in one context (in which both hormones have the same effect) and repress auxin activity in a different context (in which each hormone has the opposite effect). Distinct and overlapping types of ABA-auxin interactions raise further interesting questions about the underlying mechanisms that result in distinct tissues requiring different aspects of auxin or ABA for response to the other hormone.

We are only beginning to understand the possible points of interactions between auxin and ABA in the regulation of various plant growth and developmental processes - there is much work to be done going forward. Advances in sequencing technology, proteomics, and metabolomics now provide numerous assays that could substantially improve our understanding of how these two hormones interact in the future. Further uncovering how ABA and auxin interact to regulate growth and stress responses has strong implications for crop breeding and will be beneficial in the future.

## Figures and Tables

**Table 1:** Effects of exogenous auxin and ABA on various growth processes and their known interactions.

	Treatment Effect		
Tissue	ABA	Auxin	ABA-Auxin Interaction
Dark-grown hypocotyls	Inhibits growth [46]	Inhibits growth [47]	unknown
Light-grown hypocotyls	Inhibits growth (grown under red light) [48]	Promotes growth [49]	unknown
Dark-grown roots	unknown	Inhibits growth [50]	unknown
Light-grown roots	Inhibits growth [51]	Inhibits growth [51]	Auxin downstream [51]
Root hairs	Inhibits expansion [52]	Promotes expansion [53]	unknown
Seed germination	Inhibits [54]	Inhibits [55]	Auxin downstream [51,56]
Cotyledon expansion	Inhibits [48]	Inhibits [57]	unknown
Lateral root formation	Inhibits [58]	Promotes [59]	Auxin downstream [60,61]
